# Alpha-Synuclein Physiology and Pathology: A Perspective on Cellular Structures and Organelles

**DOI:** 10.3389/fnins.2019.01399

**Published:** 2020-01-23

**Authors:** Luis D. Bernal-Conde, Rodrigo Ramos-Acevedo, Mario A. Reyes-Hernández, Andrea J. Balbuena-Olvera, Ishbelt D. Morales-Moreno, Rubén Argüero-Sánchez, Birgitt Schüle, Magdalena Guerra-Crespo

**Affiliations:** ^1^División de Neurociencias, Instituto de Fisiología Celular, Universidad Nacional Autónoma de México, Mexico City, Mexico; ^2^Laboratorio de Medicina Regenerativa, Departamento de Cirugía, Facultad de Medicina, Universidad Nacional Autónoma de México, Mexico City, Mexico; ^3^Department of Pathology, Stanford School of Medicine, Stanford University, Stanford, CA, United States

**Keywords:** alpha-synuclein, organelle, synucleinopathies, Lewy bodies, mitochondria, nucleus, endoplasmic reticulum, Golgi apparatus

## Abstract

Alpha-synuclein (α-syn) is localized in cellular organelles of most neurons, but many of its physiological functions are only partially understood. α-syn accumulation is associated with Parkinson’s disease, dementia with Lewy bodies, and multiple system atrophy as well as other synucleinopathies; however, the exact pathomechanisms that underlie these neurodegenerative diseases remain elusive. In this review, we describe what is known about α-syn function and pathophysiological changes in different cellular structures and organelles, including what is known about its behavior as a prion-like protein. We summarize current knowledge of α-syn and its pathological forms, covering its effect on each organelle, including aggregation and toxicity in different model systems, with special interest on the mitochondria due to its relevance during the apoptotic process of dopaminergic neurons. Moreover, we explore the effect that α-syn exerts by interacting with chromatin remodeling proteins that add or remove histone marks, up-regulate its own expression, and resume the impairment that α-syn induces in vesicular traffic by interacting with the endoplasmic reticulum. We then recapitulate the events that lead to Golgi apparatus fragmentation, caused by the presence of α-syn. Finally, we report the recent findings about the accumulation of α-syn, indirectly produced by the endolysosomal system. In conclusion, many important steps into the understanding of α-syn have been made using *in vivo* and *in vitro* models; however, the time is right to start integrating observational studies with mechanistic models of α-syn interactions, in order to look at a more complete picture of the pathophysiological processes underlying α-synucleinopathies.

## Introduction

The alpha-synuclein (α-syn) protein is encoded by the *SNCA* gene localized on the long arm of chromosome 4 (Chr 4q22.1), composed of 140 amino acid residues with a molecular weight of approximately 15 kDa (Lee and Trojanowski, 2006; Bendor et al., 2013), which presents three domains. The C-terminal region is rich in acid residues (Wu et al., 2008; Oueslati et al., 2010), whereas the central region known as the non-amyloid component (NAC) permits the oligomerization of α-syn due to its hydrophobic composition (George et al., 1995; Ulmer et al., 2005). On the other hand, the N-terminal region contains four regions of 11 imperfect repeats with the KTKGEV consensus sequence. This allows the formation of an alpha helix, which enables lipid-binding; particularly, α-syn binds to negatively charged lipids (Segrest et al., 1990; Uéda et al., 1993; Bussell and Eliezer, 2003; Jao et al., 2008; Martial et al., 2019).

In its monomeric state, α-syn is intrinsically disordered and soluble, and constitutes the most common form found inside the cytoplasm (Binolfi et al., 2012; Fauvet et al., 2012; Theillet et al., 2016). The aggregation of several monomers of α-syn gives rise to oligomers, which can adopt different morphologies such as spherical, chain-like, annular (pore-like structure), and tubular (Conway et al., 2000; Ding et al., 2002; Lashuel et al., 2002). The three-dimensional structure that α-syn can adopt varies from monomers and oligomers to fibrillar conglomerates, the last two linked to cytotoxicity (Wang-Ip et al., 2017). Similarly, different mutations on the *SNCA* gene like p.A30P and p.A53T are more prone to the formation of protofibrillar intermediates of α-syn oligomers (Lashuel et al., 2002; Bengoa-Vergniory et al., 2017).

The aggregation process involving the binding between α-syn and other protein complexes such as tau protein and β-amyloid, termed “cross-seeding,” is the main mechanism for the formation of Lewy bodies (LB), which are abnormal neuronal intracytoplasmic inclusions consisting of more than 70 proteins, whose core is mainly constituted by α-syn fibrillar aggregates (Wakabayashi et al., 2007; Ono et al., 2012; Yacoubian and Standaert, 2014). This differs from the formation of fibrillar aggregates from α-syn oligomers called “seeding” (Kalia et al., 2013; Wang et al., 2016). It is thought that α-syn fibrils tend to form due to the interactions between monomers and oligomers that are thermodynamically favorable and stabilizing (Alam et al., 2019). The characteristic conformation of α-syn fibrils is β-sheets, with the individual β-strands arranged in parallel (Serpell et al., 2000; Vilar et al., 2008).

The presence and detrimental effects of abnormal cytoplasmatic α-syn accumulation, and their aberrant forms in neurons and glia are linked to neurodegenerative diseases, called synucleinopathies (Galvin et al., 2001; Martí et al., 2003), which includes Parkinson’s disease (PD), dementia with Lewy bodies (DLB), and multiple system atrophy (MSA), the former being the most common synucleinopathy (Langston et al., 2015; Sekigawa et al., 2015; Peelaerts and Baekelandt, 2016). It is notable that some *SNCA* gene allelic variants (e.g., SNCA locus triplication) are enough to develop a severe early onset PD and DLB (Singleton et al., 2003; Orme et al., 2018; Zafar et al., 2018). However, it is remarkable that up to one third of patients with PD are due to LRRK2-gene mutations/PARK8 (Kalia et al., 2015) and almost all patients with Parkin-gene mutations/PARK2 lack LB pathology (Klein and Westenberger, 2012; Poulopoulos et al., 2012).

At the cellular level, native α-syn is present in synaptic terminals, in the nucleus of neuronal cells (Maroteaux et al., 1988), mitochondria (Li et al., 2007), endoplasmic reticulum (ER) (Hoozemans et al., 2007), Golgi apparatus (GA) (Gosavi et al., 2002; Mori et al., 2002), and in the endolysosomal system (Lee et al., 2004). However, its physiological function in each subcellular compartment is only partially understood (summarized in Table 1). At the same time, the presence of the pathologic forms and its effects has been linked to both damaging and protecting outcomes, resulting in controversial conclusions using sinucleinopathies models (also summarized in Table 1). Indeed, cytotoxic effects of α-syn fibrils are related to increased oxidative stress, impaired axonal transport, ubiquitin-proteasome machinery, mitochondrial function, and synaptic dysfunction (Irwin et al., 2013; Poewe et al., 2017). These harmful intracellular effects can be facilitated by neuron-to-neuron transmission through direct penetration, synapse–synapse contact, membrane receptors, and endocytosis (Lashuel et al., 2013).

**TABLE 1:** Impairments in different organelles related to α-syn.

**Organelle**	**Impairment**	**Study model**	**Mechanism**
Mitochondria	Increased mitochondrial fragmentation	Overexpression of hWT α-syn in *C. elegans* (Kamp et al., 2010)	α-syn averts the building of fusion stalk
	Decreased protein import	(1) Lipid bilayers with recombinant monomeric α-syn (Rostovtseva et al., 2015) (2) hWT α-syn overexpression in SN of rat (Lu et al., 2013)	α-syn blocks the TOM complex and VDAC
	(A) Increased ROS production (B) Decreased ATP synthesis	(1) Overexpression of p.A53T α-syn in mouse (Chinta et al., 2010) (2) Interaction between α-syn and mitochondria in rat brain (Martínez et al., 2018) (3) Monomers and oligomers of recombinant α-syn applied to primary rat co-cultures of neurons and astrocytes (Ludtmann et al., 2018) (4) Overexpression of hWT and p.A53T α-syn in human fetal dopaminergic primary neuronal cultures (Devi et al., 2008). (5) Aggregated α-syn applied to hESC cybrids (Reeve et al., 2015). (6) Skin fibroblast with *SNCA* locus triplication (Mak et al., 2011). (7) hiPSC-derived neuronal precursor cells with *SNCA* locus triplication (Flierl et al., 2014). (8) hiPSC-derived neurons with *SNCA* locus triplication incubated with monomeric, oligomeric, and fibrillar forms of α-syn (Emma et al., 2016). (9) Brains of postmortem PD patients (Devi et al., 2008).	(A and B) The impairment in complex I decreases the electron transport chain flow, which facilitates the production of ROS, with the subsequent dissipation of the electrochemical gradient necessary for the ATP synthase function (B) ROS generated by α-syn forms generates lipid peroxidation and oxidizes the ATP synthase β subunit
	Increased mtDNA damage	hWT α-syn overexpression in mouse (Bender et al., 2006, 2013)	The increase in ROS oxidizes the mtDNA
	Increased cell death	(1) Monomers and oligomers of recombinant α-syn applied to primary rat co-cultures of neurons and astrocytes (Ludtmann et al., 2018) (2) hiPSC-derived neuronal precursor cells with *SNCA* locus triplication (Flierl et al., 2014)	α-syn interacts directly with permeability transition pore components and decreases their threshold opening
Nucleus	Increased stiff and length of DNA	Nanofluidic system with DNA from phage lambda and α-syn (Jiang et al., 2018).	Through binding to naked DNA.
	Impaired DNA methylation	Transgenic mice expressing hWT α-syn under the Thy-1 promoter, rat B103 neuroblastoma cells and 293T human hepatocarcinoma cells (Desplats et al., 2011)	α-syn retains DNA methyltransferase 1 in the cytoplasm
	Impaired histone deacetylation	SH-SY5Y cells with hWT α-syn expression (Kontopoulos et al., 2006)	α-syn restricts and maintains histone deacetylases in the cytoplasm
	Alteration in histone methylation pattern	*D. melanogaster* expressing hWT α-syn ubiquitously under control of a daG32-GAL4 driver and dopaminergic differentiated SH-SY5Y cells with inducible hWT α-syn expression (Sugeno et al., 2016)	α-syn selectively enhances H3K9 mono- and dimethylation by interacting with H3K9me1/2 methyltransferase
Endoplasmic Reticulum (ER)	Increased ER stress and cellular death	(1) hiPSC-derived cortical neurons overexpressing α-syn due to *SNCA* locus 3 (Heman-Ackah et al., 2017) (2) SH-SY5Y cell expressing pS129-α-syn (Sugeno et al., 2008) (3) LUHMES cells and mice expressing p.A30P α-syn (Paiva et al., 2018)	(1) UPR activated by induction of inositol-requiring transmembrane kinase/endoribonuclease 1α/X-box binding protein 1 pathway (2) Overactivation of UPR by induction of ER stress (3) Increasing level of ER stress with *COL4A2* gen up-regulated
	Impairment in calcium homeostasis	(1) PC-12 cell line overexpressing p.A30P or p.A53T α-syn mutant (Smith et al., 2005) (2) Mouse dopaminergic cell line (CATH.a) overexpressing hWT α-syn (Yoon et al., 2018) (3) BE(2)-M17 neuroblastoma cells expressing pA53T or p.A30P (Guardia-Laguarta) (4) Transgenic mutant p.A53T α-syn mice (Belal et al., 2012)	(1) Overexpression of ER stress markers (78-kDa glucose-regulated protein, inositol-requiring enzyme 1 and phosphorylated eukaryotic initiation factor 2α) (2) Induction of ER stress by exposure to manganese (3) α-syn relocation from cytoplasm to the vicinity of mitochondrial-associated ER membranes (4) Induction of ER stress by action of homocysteine-induced ER protein
	Increased cell apoptosis	(1) SH-SY5Y cells expressing hWT α-syn (Betzer et al., 2018)	(1) Activation of sarco/ER Ca^2+^-ATPase
	Aberrant vesicular traffic	(1) *S. cerevisiae* with null expression of *ELO1*, *ELO2* and *ELO3* concomitant to the expression of hWT α-syn, A53T or E46K (Lee et al., 2011) (2) hiPSC-derived midbrain dopamine neurons overexpressing hWT α-syn (Mazzulli et al., 2016)	(1) Accumulation of ROS within ER (2) Diffuse localization of Rab1A with ER-GA fragmentation
Golgi apparatus (GA)	Increased GA fragmentation	(1) Primary rat astrocytes with overexpression of WT α-syn or A30P or A53T mutants (Liu et al., 2018) (2) LUHMES cells and mice expressing p.A30P α-syn (Paiva et al., 2018) (3) COS-7 that expressed α-syn (Gosavi et al., 2002) (4) Nigral neurons from patients with PD (Fujita et al., 2006)	(1) Activation of the transcription factor CCAAT-enhancer-binding protein homologous protein (2) Indirect mechanism by increasing level of ER stress with *COL4A2* gen up-regulated (3) Accumulation of α-syn and presence of its fibrillary form (4) Unknown
	Increased cell death	*S. cerevisiae*, *C. elegans*, and *Drosophila melanogaster* expressing hWT α-syn (Büttner et al., 2012)	Increasing calcium level by Ca^2+^/Mn^2+^-transporting P-type ATPase activation
	Impairment in ER-GA transport	(1) hESC-derived astrocytes with exposure to recombinant α-syn oligomers (Rostami et al., 2017) (2) SKNSH human neuroblastoma cells with overexpressed α-syn (Winslow et al., 2010) (3) Overexpression of hWT α-syn and A53T in a yeast model (Cooper et al., 2006) (4) Rat kidney epithelial and rat PC-12 cells overexpressing hWT and mutant p.A53T α-syn (Thayanidhi et al., 2010)	(1) Oligomers accumulation in lysosome and disruption of autophagosome/lysosome (2) Accumulation of α-syn inhibits autophagosome formation in form dependent of Rab1A (3) Cytoplasmic inclusion of α-syn suppresses ER-GA transport (4) Antagonism of soluble N-ethylmaleimide-sensitive factor attachment protein receptor and inhibition of docking and fusion of vesicles covered with coat protein complex II
	Vesicular traffic alteration and Golgi post-translational modifications	(1) *S. cerevisiae* that express hWT α-syn with a vector (Willingham et al., 2003) (2) *S. cerevisiae* with inducible expression of hWT α-syn or A53T mutant (Soper et al., 2008). (3) SH-SY5Y neuroblastoma cells with expression of α-syn (Lee et al., 2006) (4) *S. cerevisiae* with expression of hWT α-syn (Soper et al., 2011)	(1) Dysregulation of gene expression (*glo4, mal31* or *tlg2*) and alteration in GTPases homeostasis (2) Accumulation of membranous vesicles with nearness of α-syn (3) Impairment of microtubule-dependent trafficking by overexpression of α-synuclein (4) Induction of accumulation and mislocalization of Rab proteins

For the most part, the stimuli that trigger oligomer formation are unknown, although several events have been characterized that seem to favor their appearance, such as an increase in temperature and a decrease in pH (Uversky et al., 2001). In addition, the incubation of 4-hydroxy-2-non-enal, a product of lipid oxidation and related to neurodegenerative diseases, with human wild-type (hWT) α-syn, prevents its fibrillation but induce β-sheet-rich oligomers (Qin et al., 2007). Similar results can be obtained in mesencephalic neuronal cells cultures exposed to polyunsaturated fatty acids (Sharon et al., 2003). It is suggested that the main route by which oligomers are cytotoxic is by disruption (pore formation) of the plasma membrane of the cells (Tsigelny et al., 2012; Froula et al., 2019). These pores facilitate the diffusion of molecules and ions, especially Ca^2+^, which, in high concentrations, is harmful to neurons, inducing cell death (Quist et al., 2005; Angelova et al., 2016; Fusco et al., 2017).

Another relevant conformation of α-syn is given by its phosphorylation. Furthermore, the main post-translational modification of α-syn in LB is that more than 90% is phosphorylated in the serine 129 residue (pS129-α-syn), while brains without LB represent less than 4% of total α-syn (Fujiwara et al., 2002; Anderson et al., 2006). Although the kinase responsible for this phosphorylation in the context of LB formation is unknown, several candidate kinases have been proposed to phosphorylate a-syn, including casein kinase 2 (CK2), G protein-coupled receptor kinase 2 (Gprk2), and Polo-like kinase 2 (PLK2).

It has been found *in vitro* that CK2, a serine/threonine kinase, may be responsible for cytosolic α-syn and membrane-associated α-syn phosphorylation (Hara et al., 2013), since in 3D5 cells exposed to ferrous chloride, there was an increment in α-syn inclusions, along with CK2 up-regulation. Furthermore, the inhibition of this kinase decreased the formation of such inclusions (Takahashi et al., 2007).

In parallel, in a *Drosophila* model of PD, the expression of hWT α-syn and Gprk2, which has α-syn as a substrate (Pronin et al., 2000), leads to an increment of pS129-α-syn and α-syn-mediated toxicity was observed (Chen and Feany, 2005). There is also evidence that Gprk family mediated phosphorylation occurs only in membrane-associated α-syn (Hara et al., 2013).

Besides phosphorylation of the serine 129 residue mediated by PLK2, a serine/threonine kinase could also be involved in a mechanism of α-syn clearance by the lysosome–autophagic degradation pathway. PLK2 overexpression in HEK cells decreases the amount of α-syn that correlates with the amount of PLK2 and pS129-α-syn. Also, when 3MA (a lysosomal pathway blocker) is added, the amount of α-syn returns to baseline levels (Oueslati et al., 2013).

Among the functions attributable to pS129-α-syn are as a regulator of dopamine uptake; in SH-SY5Y and HEK293 cells, the membrane-bound pS129-α-syn is associated with increased expression of DAT on the cell surface (Hara et al., 2013). Furthermore, the phosphorylation of pS129-α-syn might also be a mechanism whereby the cell reduces the damage caused by the formation of pores caused by alpha-synuclein oligomers. In unilamellar lipid vesicles, it has been shown that pS129-α-syn inhibits the binding of α-syn oligomers to the membrane and therefore decreases the pore formation (Nübling et al., 2014).

Despite this evidence, it remains to be clarified what is the role of pS129-α-syn. It seems that in early stages of α-syn accumulation and formation of α-syn oligomers, the pS129-α-syn may act like a compensatory mechanism, but in later stages, pS129-α-syn could mediate neurotoxic events. Furthermore, the phosphorylation observed in fibrils might be a result, not a catalyst, of the aggregation process (Paleologou et al., 2008). For these reasons, it has been postulated that LB could have a cytoprotective role; however, current evidence is still controversial (Tanaka et al., 2004; Lázaro et al., 2014).

In this review, we discuss current evidence related to α-syn as a prion-like protein, the interactions between α-syn with soluble and insoluble proteins, as well as the function of α-syn within different organelles. We also include data on human-induced pluripotent stem cells (hiPSC), derived from PD patients, since α-syn is a ubiquitous protein that, when it is present in monomeric form, performs physiological functions (e.g., regulation of mitochondrial fusion–fission), but when grouped in toxic conformations (e.g., oligomers or fibrils) is related to organelle dysfunction (e.g., mitochondrial and GA fragmentation) that ultimately lead to pathological processes.

## Prion-Like Characteristics of α-Syn

A prion is a protein with aberrant folding, inducing aggregation by physically interacting with other proteins. Through this process, it generates a great quantity of misfolded proteins, which are pathological for the carrier (patient). Nevertheless, a fundamental characteristic of prion proteins is their inter-organism transmissibility, causing the same phenotype, making prions infectious agents with high morbidity (Prusiner, 1998; Geschwind, 2015). The fact that α-syn aggregates have been found in different regions of the brains of PD patients depending of the disease stage (Braak et al., 2003), evidence of α-syn propagation from the gut to the brain (Kim et al., 2019), and the finding of LB in grafted fetal midbrain neurons into the striatum of PD patients over 10 years after transplantation (Kordower et al., 2008; Li et al., 2008) have led several groups of researchers to conclude that α-syn acts as a prion-like protein (Angot et al., 2010; Brundin et al., 2010, 2016; Dunning et al., 2012, 2013; Tomé et al., 2013; Surmeier et al., 2017). But so far, no evidence has been found supporting its transmissibility from one organism to another. Therefore, in PD context, α-syn cannot be tagged as a prion protein, but it can be studied more as a prionoid or prion-like protein (Aguzzi, 2009; Scheckel and Aguzzi, 2018). Interestingly, MSA, another synucleinopathy, is classified as a prion disease due to the evidence that inoculation of samples from brains of MSA patients into p.A53T α-syn transgenic mice brains causes aggregation and spreading of α-syn, in addition to a MSA-like phenotype. Indeed, these results can be replicated by inoculating homogenate from the first transplanted rat brain into a second rat brain (Prusiner et al., 2015). Similar results can be found when the rat is exposed peripherally (like peritoneal cavity) to the homogenates of MSA brains (Woerman et al., 2018). Nevertheless, some issues need to be taken into account, like the fact that these results could not be obtained in wild-type (WT) mice (Prusiner et al., 2015). In relation to DLB, more information is needed to know if it has a transmission mechanism like prions.

## Alpha-Synuclein Interactions

As mentioned earlier, α-syn contains an N-terminal domain, which permits its interaction with membrane lipids (Uéda et al., 1993), and both the pS129-α-syn and α-syn phosphorylated at tyrosine 125 (pY125-α-syn) represent pathological forms of α-syn (Okochi et al., 2000; Ellis et al., 2001; Takahashi et al., 2002). In order to unravel the interactions of phosphorylated and non-phosphorylated α-syn, McFarland et al. (2008) performed a comparative proteomic approach. Peptides containing 40 amino acids of the C-terminal domain of hWT α-syn and a phosphomimic form, which consisted in the substitution of the serine 129 for aspartic acid, were used. Mass spectrometric analysis of solubilized mouse brain synaptosome proteins, pulled down with the peptides, allowed the observation that the phosphorylated α-syn peptide could interact with cytoskeletal and vesicular traffic proteins, as well as serine protein kinases, whereas non-phosphorylated α-syn could not (McFarland et al., 2008). On the contrary, with the same peptides, non-phosphorylated α-syn showed interaction with oxidative phosphorylation (OXPHOS) proteins, but this interaction was not observed for phosphorylated α-syn (McFarland et al., 2008).

**FIGURE 1:**
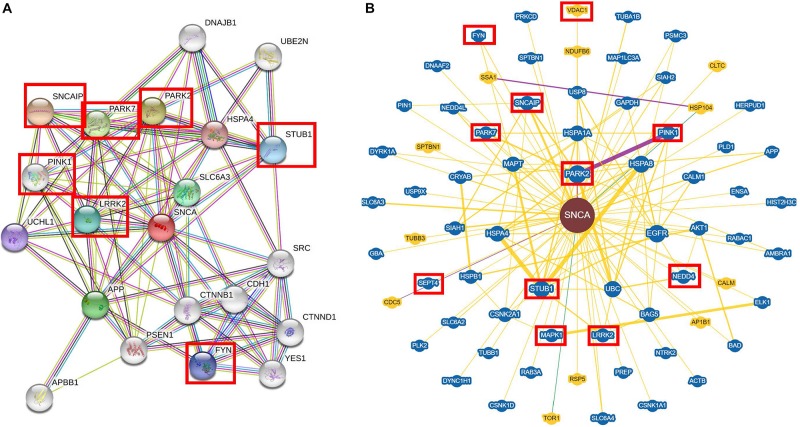
Interaction of the alpha-synuclein with soluble proteins by STRING and BioGRID^3^.^5^. **(A)** STRING and **(B)** BioGRID^3^.^5^ show that alpha-synuclein (α-syn) interacts mostly with kinase proteins like LRKK2, MAPK1, Fyn, etc. and it can interact with ubiquitin proteins like PARK2, PARK7, STUB1, etc. Each interactor is shown as a circle and the lines represent the interaction between the proteins. The interactors in a red box are mentioned in the interaction section and also throughout the review.

To highlight the multiple interactions of α-syn, we used a platform of predicted interactions of proteins (STRING, Szklarczyk et al., 2017) and a dataset of reported information from individual studies (BioGRID^3^.^5^, Oughtred et al., 2016). For the STRING analysis (Figure 1A), we used *SNCA* as a key word and *Homo sapiens* as organism of search. These analyses were performed establishing 20 interactors instead of the 10 showed by default in the program, in order to have a greater landscape of its interactions and 0.400 was used as the interaction score, which is a threshold on the confidence score, such that only interactions above this score were included in the network. The STRING analysis showed that α-syn presented greater interaction with synphilin-1, which has been reported to bind to vesicle membranes of the presynaptic terminals and interacts with α-syn to inhibit the docking of the fusion vesicles. This suggests that synphilin-1 mediates the synaptic function of α-syn in HEK293 cells (Krüger, 2004; Szargel et al., 2008). The interaction between synphilin-1 and α-syn was also observed in a yeast model with the same effect of inhibition in the docking of the fusion vesicles; however, the function of synphilin-1 needs further research (Neystat et al., 2002) (Figure 1A). In addition, in our analyses, α-syn interacts with kinase proteins like LRKK2 (Harvey and Outeiro, 2018) and Fyn (Nakamura et al., 2001), as well as ubiquitin ligases like PARK2 and PARK7 (Choi et al., 2001; Hauser et al., 2017) and STUB1 (Kalia et al., 2011), which are reported to be involved in PD.

For the BioGRID^3^.^5^ analyses (Figure 1B), we used *SNCA* as a keyword and *H. sapiens* as organism of search. In the BioGRID^3^.^5^ analyzes, it was found that α-syn showed 69 interactor proteins, which included kinase proteins such as Pink1, involved in mitochondrial function (Cookson, 2012); LRKK2, which mediates assembly of the cytoskeleton (Harvey and Outeiro, 2018); MAPK1, implicated in many cell functions like cell adhesion, cell cycle progression, cell differentiation, among others (Roskoski, 2012; Bohush et al., 2018); and SEPT4, which is a protein with a nucleotide-binding domain that regulates cytoskeletal organization (Ihara et al., 2007). Other proteins that interact with α-syn are ubiquitin ligases like STUB1, involved in cell death regulation (Kalia et al., 2011); Park2, which ubiquitinates Bax protein in mitochondria and regulates apoptosis (Hauser et al., 2017); and NEDD4, which ubiquitinates α-syn to lead its degradation (Chung et al., 2013). Extensive reviews about experimental evidence of the interaction between α-syn and proteins can be found elsewhere (Emamzadeh, 2016; Khurana-Langs et al., 2017).

**FIGURE 2:**
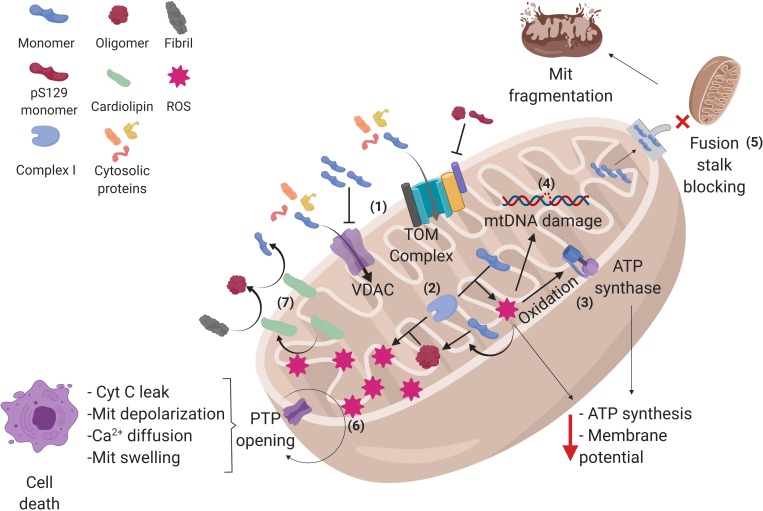
Alpha-synuclein conformations and its interactions in mitochondria. (1) Alpha-synuclein (α-syn) monomers enter the mitochondria (Mit) through translocase of the outer membrane (TOM) and voltage-dependent anion channels (VDAC), but oligomers and pS129-α-syn on TOM and α-syn accumulation on VDAC can inhibit the protein import into the mitochondria, affecting the process that depends on cytosolic proteins. (2) Once inside, α-syn can interact with complex I of the electron transport chain, raising reactive oxygen species (ROS) production within the mitochondria, and favoring the aggregation of α-syn monomers into oligomers, which in turn produces more ROS, creating a cycle where α-syn aggregation and ROS production exacerbate each other. (3) The increased amount of ROS oxidizes the ATP synthase β-subunit, diminishing mitochondrial ATP levels as well as (4) damaging mitochondrial DNA (mtDNA), which may lead to an altered expression of mitochondrial genome-encoded genes. (5) α-syn monomers can also block mitochondrial fusion stalk, producing mitochondrial fragmentation. (6) In addition, α-syn induces the opening of permeability transition pore (PTP), allowing cytochrome C (cyt C) leak, depolarization, calcium diffusion, and swelling of the mitochondria, leading to cell apoptosis. (7) Certain stress events such as ROS can stimulate the translocation of cardiolipin from the inner membrane to the outer membrane, where it acts as a buffer for aggregative synuclein forms. See the text for further details. Created with BioRender.

**FIGURE 3:**
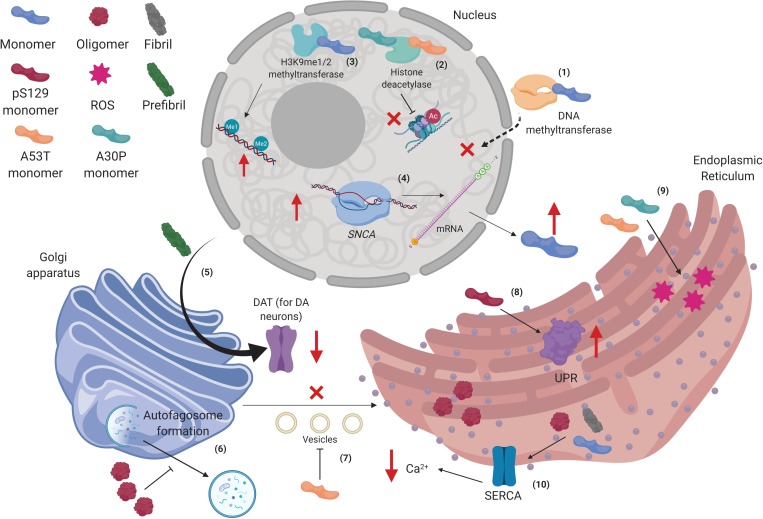
Alpha-synuclein interactions in the nucleus, Golgi apparatus, and the endoplasmic reticulum. (1) Human wild type alpha-synuclein (hWT α-syn) can retain methyltransferases in the cytoplasm, thus altering DNA methylation of the *SNCA* gene. (2) hWT α-syn can also interact with H3K9me1/2 to increase mono- and dimethylation in the DNA. (3) p.A53T and p.A30P monomers bind to HDACS and inhibit histone deacetylation. (4) Decreased epigenetic regulation on the *SNCA* gene promotes its up-regulation, increasing α-syn transcription and further accumulation. (5) Prefibrillar α-syn can disrupt postraductional processing of dopamine transporter (DAT) in the Golgi apparatus (GA), diminishing its presence in the membrane. (6) α-syn oligomers impair autophagosome formation in the GA. (7) p.A53T monomers inhibit vesicles transport from endoplasmic reticulum (ER) to GA. (8) pS129-α-syn monomers increase unfolded protein response (UPR) activity in the ER. (9) p.A53T and p.A30P monomers increase levels of stress in the ER. (10) α-syn oligomers and fibrils affect SERCA complex in the ER, diminishing cytosolic levels of Ca^2+^. Created with BioRender.

The closest interactions to α-syn found in both STRING and BioGRID^3^.^5^ analyses showed soluble protein interactors that are involved in cellular process such as degradation (Figure 1). However, it has been determined that α-syn can also bind to insoluble proteins, which are localized in membranous compartments of the cells. Furthermore, some of these proteins are voltage-dependent anion channel (VDAC), translocase of the outer membrane 20 (Tom20), and adenylate translocator in mitochondria (Figure 2) (Zhu et al., 2011; Lu et al., 2013; Di Maio et al., 2016). α-syn can also be localized with importin alpha in the nucleus (Figure 3) (Ma et al., 2014), sarco/ER Ca^2+^-ATPase (SERCA) in the ER (Figure 3) (Betzer et al., 2018), and Rab1A both in the ER and the endolysosomal system (Mazzulli et al., 2016) (Figure 3). The interactions between α-syn and the proteins mentioned are described below.

## Alpha-Synuclein Interacts Both Physiologically and Pathologically With Mitochondrial Components

The mitochondria is a double-membrane-bound organelle involved in oxidation of metabolites, generation of adenosine triphosphate (ATP) through OXPHOS, Ca^2+^ signaling, and apoptosis (Hock and Kralli, 2009). α-syn has a higher affinity for mitochondrial membranes compared to lipid membranes of the other organelles (Nakamura et al., 2008; Kamp et al., 2010). α-syn presence was determined by Western blot in the isolated mitochondrial and cytosolic fractions of WT rat brains (Li et al., 2007), whereas in humans, the relationship between α-syn with mitochondria was determined due to the pathological findings of α-syn accumulation within the mitochondria of brain regions like the substantia nigra (SN) and the striatum of PD patients (Devi et al., 2008), although mitochondrial dysfunction was a well-known phenomenon in PD since the 1980s (Schapira et al., 1989).

Specifically, α-syn has been reported in the three main mitochondrial structures: the inner mitochondrial membrane (IMM), the outer mitochondrial membrane (OMM), and the mitochondrial matrix (MM) (Cole et al., 2008; Devi et al., 2008; Liu et al., 2009; Kamp et al., 2010; Robotta et al., 2014). Interestingly, pS129-α-syn has a higher binding affinity to the OMM than the WT α-syn (Wang et al., 2019). However, the same is not applicable for the ER and the GA (Ryan et al., 2018).

The first 32 amino acids of the N-terminal of the α-syn are necessary for its translocation to mitochondria (Devi et al., 2008) and α-syn can be internalized without the need of post-translational modifications or that has been mobilized first through another organelle (Martínez et al., 2018). It has a role as a regulator in some of the mitochondrial physiological functions like mitochondrial fusion, electron transport chain (ETC), and VDAC permeability, among others (Figure 2) (Ellis et al., 2005; Kamp et al., 2010; Rostovtseva et al., 2015).

Seo et al. (2002) showed results of a protective role with low and physiological concentrations of α-syn (0.1–1.0 μM) against oxidative stress in cortical and hippocampal neurons of rats and cell cultures of LHRH (luteinizing hormone-releasing hormone)-secreting neurons, SH-SY5Y neuroblastoma cells, and pheochromocytoma (PC-12) cells. Nevertheless, α-syn accumulation, overexpression, or impairment of its degradation mediates mitochondrial dysfunction that can lead to more α-syn accumulation, misfolding, and inclusions (Hsu et al., 2000; Lee et al., 2002; Chinta et al., 2010). Although the molecular mechanisms of these phenomena are not completely understood, it is postulated that α-syn oligomers form covalent cross-links between tyrosine residues under oxidative conditions, giving rise to larger filaments, and these α-syn conformations can increase reactive oxidative species (ROS) production by interacting with ETC elements, resulting in a cycle where the aggregation and generation of ROS exacerbates each other (Figure 2) (Hashimoto et al., 1999; Souza et al., 2000; Scudamore and Ciossek, 2018).

Related with the above, the overexpression of hWT α-syn in *Caenorhabditis elegans* body wall muscle accelerated age-related mitochondrial morphological changes (Table 1) (Kamp et al., 2010). Furthermore, in mice overexpressing hWT α-syn, mitochondrial dysfunction in dopaminergic neurons from the nigrostriatal pathway preceded several months before depletion of striatal dopamine (Lam et al., 2011; Subramaniam et al., 2014).

### Outer Mitochondrial Membrane

#### Alpha-Synuclein Is Associated With Mitochondrial Morphological Changes

Mitochondrial fusion is a process by which mitochondria can mix their content, homogenizing it and avoiding loss of essential material like mitochondrial DNA (mtDNA) localized in the MM. Impairment of mitochondrial fusion is linked to neurodegenerative diseases (Chen and Chan, 2010). A recent study found that the overexpression of α-syn in neurons can confer protection against ROS, since it decreases a type of dynamin-related protein 1 (Drp1)-dependent mitochondrial fragmentation related to H_2_O_2_ and 6-hydroxydopamine, known as mitosphere (hyperpolarized mitochondria with a spherical form), which precedes the activation of apoptosis mediated by Caspase 3 (Menges et al., 2017). However, further research is needed in order to know the α-syn baseline concentration contribution in mesencephalic dopaminergic neurons, in order to understand the importance of α-syn in the most affected cell type, which indeed has a high rate of ROS.

Alpha-synuclein could, in part, act like a physiological regulator of mitochondrial fusion. Indeed, mitochondria need to be smaller in size (fission) in order to be mobilized through axons of dopaminergic neurons, as they have a high energy expenditure and therefore need a large number of mitochondria in synaptic terminals. Recent evidence suggests that α-syn inhibits mitochondrial fusion proteins like mitofusin-1 (Mfn1), mitofusin-2 (Mfn2), and optic atrophy type 1 (Opa1). Furthermore, α-syn binds to curved membrane sites on the OMM, blocking the fusion stalk buildup, a necessary structure for mitochondrial fusion, thus favoring mitochondrial fission. However, α-syn accumulation inhibits mitochondrial fusion proteins and lowers the proportion of fusion stalks that form correctly, hence promoting mitochondrial fragmentation (Figure 2) (Kamp et al., 2010; Menges et al., 2017; Pozo Devoto and Falzone, 2017).

In agreement with the above, HeLa cells overexpressing α-syn exhibit increased mitochondrial fragmentation (independent of intrinsic fusion proteins, e.g., Mfn1, Mfn2, Opa1 and fission proteins, e.g., Drp1) that preceded mitochondrial function impairment (Nakamura et al., 2011). Similar results were found in SH−SY5Y cells expressing hWT or p.A53T α-syn, but the same research described that both spinal cord and cortex neurons of mice that express mutant p.A53T α-syn also had an age−dependent manner of mitochondrial morphological changes (e.g., decreased of mitochondrial connectivity, related to mitochondrial fragmentation and increased in circularity, which is in turn linked to reduced mitochondrial length) together with a decrease in fusion–fission proteins. The impairment was attributable to Mfn2 decrease, since expression of this protein in SH−SY5Y cells rescued the phenotype (Xie and Chung, 2012). These results suggest that WT and mutated forms of α-syn have similar implications on mitochondrial morphology, but through different mechanisms.

#### Alpha-Synuclein Can Prevent Cytosolic Protein Import by Blocking the Translocase of the Outer Membrane (TOM) and Voltage-Dependent Anion Channel

Although mitochondria have their own DNA, they only contain genetic coding information for 13 proteins, and most mitochondrial proteins are nuclear encoded. Thus, mitochondria need to import more than 99% proteins from the cytoplasm for its function (Friedman and Nunnari, 2014), which is facilitated by transport systems like TOM, a protein complex located in the OMM, allowing the translocation of proteins from the cytosol to the mitochondrial intermembrane space. TOM is composed of seven proteins (Tom5–7, Tom20, Tom22, Tom40, and Tom70) (Sherman et al., 2005), and the proteins to be translocated to the mitochondrial intermembrane space contain specific mitochondrial targeting signal (MTS) recognized by Tom20 and Tom22, whereas Tom40 forms a β-barrel protein-conducting channel (Becker et al., 2008).

Recent studies using the *in situ* proximity ligation assay (PLA) in SH-SY5Y cell cultures and fluorescence resonance energy transfer (FRET) in rat brain mitochondria have shown that small oligomers of α-syn, pS129-α-syn, and dopamine-modified α-syn interact with the MTS recognition site on Tom20, blocking the Tom20–Tom22 interaction, necessary for protein import, resulting in diminished protein import. In contrast, monomeric α-syn, nitrated α-syn, and thioflavin T–positive α-syn fibrils do not affect the TOM system (Di Maio et al., 2016; Martínez et al., 2018). The impairment in protein import has consequences for key mitochondrial proteins imported from the cytosol, for example, the lack of NADH:ubiquinone oxidoreductase core subunit S3 (Ndufs3), a complex I subunit of the ETC, led to metabolic deficits and excessive production of ROS (Figure 2) (Suhane et al., 2013). This phenotype could be prevented by Tom20 overexpression (Di Maio et al., 2016).

Reduced levels of Tom40 have also been found in hWT α-syn overexpression transgenic mice models and in the midbrain of postmortem patients with PD; similar to Tom20, Tom40 overexpression in mice decreased α-syn-induced mitochondrial dysfunction (Bender et al., 2013). It is noteworthy that, when α-syn is phosphorylated (pS129-α-syn), it reduces its interaction with Tom40 (McFarland et al., 2008; Vicario et al., 2018).

On the other hand, VDAC is a pore with a β-barrel structure located in the OMM. It accounts for about 10% of the OMM total protein and it allows the diffusion of ions, metabolites, and polypeptides in an alternated open/close state voltage-dependent manner (Mazure, 2017). Like the TOM complex, there is evidence demonstrating that α-syn interacts reversibly with VDAC in a concentration-voltage-dependent manner (Figure 1); besides, VDAC can translocate α-syn into the mitochondria, where it can interact with complex I of the ETC and contribute to mitochondrial dysfunction (Lu et al., 2013; Rostovtseva et al., 2015). At nanomolar concentrations (50 nm) of α-syn, its negatively charged C-terminal region interacts with the positively charged pore of VDAC, creating a steric block for flow through the pore. For example, the ATP/ADP exchange between cytosol and intermembrane space could be altered, resulting in diminished ATP synthase activity, decreased membrane potential, and impairment of OXPHOS (Rostovtseva et al., 2015) (Figure 2).

Therefore, the alterations caused by the α-syn on TOM complex (prevent the binding of MTS to Tom20) and VDAC (steric pore blockage), the most important systems in the translocation of cytoplasmic proteins to the mitochondria, have repercussions both in bioenergetics and in the maintenance of structures of this organelle, which can contribute to mitochondrial dysfunction that are seen in synucleinopathies.

#### Cardiolipin Has the Capacity to Buffer Alpha-Synuclein Toxic Forms

Cardiolipin is an anionic phospholipid formed by the union of two phosphatidylglycerol molecules with a glycerol molecule (Pennington et al., 2019) located mainly in the bacterial plasmatic membrane and in the IMM (Paradies et al., 2014). It is well established that α-syn can interact with cardiolipin (Cole et al., 2008) through its N-terminal KAKEGVVAAAE repeats, specifically with cardiolipin acyl side chains via electrostatic interactions (Zigoneanu et al., 2012). The OMM has low concentrations of cardiolipin; however, externalization of cardiolipin from the IMM is stimulated under stress conditions, mitochondrial morphology alterations, pro-mitophagy conditions, and low pH (Cole et al., 2008; Nakamura et al., 2011; Chu et al., 2013), where it is necessary for (1) the recruitment of LC3 (autophagy protein microtubule-associated-protein-1 light chain 3), a required protein for the binding of the phagophore membrane to the impairing mitochondria, and (2) the binding of pro-apoptotic molecules like caspase-8, Bax, Bak, and tBid (Chu et al., 2013; Dudek, 2017). α-syn may regulate the LC3-induced mitophagy as they compete for the same binding site on such lipid (Ryan et al., 2018).

The same study using hiPSC carrying an SNCA p.A53T mutation and its generated gene-corrected isogenic hiPSC line has shown that cardiolipin in the OMM can bind to mutant α-syn to fold it into a α-helix conformation. Similarly, cardiolipin reverses α-syn oligomeric fibrils in aggregated β-sheet α-syn monomers, and from this conformation, it can turn into α-syn monomers with α-helix structure (Figure 2). This allows mitochondria to have the capacity to buffer toxic forms of α-syn (Ryan et al., 2018). However, recent evidence shows that α-syn oligomers have a greater propensity to form pores in cardiolipin-rich membranes, which could contribute to the mitochondrial dysfunction (Ghio et al., 2019).

These findings demonstrate that, through the translocation of cardiolipin, mitochondria are able to buffer the pathological forms of α-syn. However, it can also have a detrimental effect because it facilitates the formation of membrane pores that can lead to mitochondrial dysfunction. Therefore, further studies are needed to determine which effect has a greater preponderance.

### Inner Mitochondrial Membrane – Mitochondrial Matrix

In mitochondria, the IMM has a high α-syn concentration determined by immunoelectron microscopy and Western blot analysis (Devi et al., 2008; Liu et al., 2009), to which it binds in an α-helical conformation (Robotta et al., 2014). The high concentration of α-syn in the IMM may be due to the cardiolipin content in this structure (Dudek, 2017) and the fact that α-syn interacts with many components of the ETC–OXPHOS.

#### The Pathological Interaction Between Complex I of the ETC and Alpha-Synuclein Leads to Mitochondrial Dysfunction

The ETC is formed by four complexes (CI–CIV) located in the IMM, whose function is the coupling between the transport of electrons between the complexes and the pumping of protons, creating an electrochemical gradient between the mitochondrial matrix and the intermembrane space, for the synthesis of ATP by the ATP synthase (Cogliati et al., 2018). Complex I or NADH:ubiquinone oxidoreductase accepts the electrons from NADH and delivers it to Complex III through the electron-carrier ubiquinone. It also pumps protons from the mitochondrial matrix to intermembrane space (Busch, 2018; Cogliati et al., 2018).

Evidences that link α-syn physiological role to ETC is that, first, α-syn knockdown cells have a concentration and activity reduction in NADH cytochrome C reductase, an event related to complex I/III activities, which may be due to a change in lipid metabolism caused by the α-syn deficiency (Ellis et al., 2005; Devi et al., 2008), and second, the fact that triple synuclein knockout (α-, β-, and γ-syn) is resistant against complex I inhibitor rotenone (Zharikov et al., 2015). It has also been proposed that detrimental effects (e.g., increased in ROS production, decreased ATP synthesis) on complex I activity are due to an exacerbation of their likely physiological role (not yet elucidated) as a negative regulator of complex I, which eventually leads to mitochondrial impairment (Figure 2) (Loeb et al., 2010).

Accordingly, the pathological effects of α-syn causes down-regulation of complex I activity, which in turn lead to ETC impairment (especially in ventral midbrain) (Mak et al., 2011; Rostovtseva et al., 2015; Martínez et al., 2018), decreased ATP synthesis (Flierl et al., 2014), and increased mitophagy and ROS production (mainly superoxide and H_2_O_2_), a well-known promoter of α-syn oligomerization (Reeve et al., 2015; Emma et al., 2016) (Figure 2).

The fact that the presence and accumulation of α-syn (either hWT or pathological forms) lead to alterations carried out by the complex I makes it an important element of mitochondrial dysfunction, due to the fact that it can trigger and perpetuate other events harmful to the mitochondria and the cell (e.g., cycles where α-syn favors the production of ROS and these favor the aggregative forms of α-syn and vice versa). Therefore, complex I can be an element to consider as a therapeutic target.

#### mtDNA Damage Is Associated With Alpha-Synuclein Overexpression and Parkinson’s Disease

The ROS production by overexpression of hWT α-syn in mouse models has been postulated as one of the causes of mtDNA damage, especially somatic mtDNA deletions that may lead to an altered expression of mitochondrial genome-encoded genes, like complex I and complex IV subunits, which would increase the damage by ETC and OXPHOS (Bender et al., 2006, 2013) (Figure 2). There is also evidence that the prevalence of mtDNA mutations increases with age, predominantly in vulnerable cells like dopaminergic neurons and regions like caudate and putamen (in comparison to cells from cerebral cortex, cerebellum, and dentate nucleus), and are higher in PD patients (Soong et al., 1992; Bender et al., 2006; Kraytsberg et al., 2006). As mentioned above, mtDNA mutations are related to low expression of mtDNA-encoded proteins (e.g., subunits of complex IV) (Bender et al., 2006; Kraytsberg et al., 2006). Interestingly, the number of copies of mtDNA increases with age, which is thought to be a compensatory mechanism to age-related mutations in mtDNA. However, this mechanism is diminished in patients with PD (Dölle et al., 2016).

#### The Interaction of Alpha-Synuclein With ATP Synthase Is Dependent on Their Aggregation State

Adenosine triphosphate synthase uses the electrochemical gradient generated by the proton pumping of the ETC for the phosphorylation of ADP to form ATP (Cogliati et al., 2018). It has also been shown through PLA assays that α-syn monomers interact with the ATP synthase α-subunit, which suggests that α-syn acts as a regulator of the ATP synthase. Triple α-, β-, and γ-syn knockout midbrain neuronal cells of mice have lower mitochondrial membrane potential and uncoupling between OXPHOS and respiration, which leads to a decrease in ATP synthase activity due to low respiratory control ratio values. Monomeric α-syn rescues this phenotype (Ludtmann et al., 2016). In contrast, another study found that α-syn knockout mouse neurons do not exhibit alterations in mitochondrial bioenergetics (Pathak et al., 2017).

On the other hand, α-syn oligomers have an opposite effect on ATP synthesis. The ROS produced by these toxic forms of α-syn generate lipid peroxidation and oxidize the ATP synthase β subunit, which impairs its function, thus leading to alterations in OXPHOS, due to the depolarization of the membrane. These oxidative events can stimulate the opening of the permeability transition pore (PTP) (Ludtmann et al., 2018).

#### Alpha-Synuclein Can Lead to Cell Death Through Stimulation of PTP

The hWT α-syn interacts directly with an adenylate translocator (Zhu et al., 2011) and VDAC (Lu et al., 2013; Rostovtseva et al., 2015), both part of the PTP. The opening of the PTP allows the diffusion of molecules of <1.5 kDa and ions, including Ca^2+^, resulting in membrane depolarization and mitochondrial swelling that leads to OMM rupture with concomitant cytochrome C leak, events that trigger cell death (Halestrap, 2009; Luth et al., 2014). In the same manner, hiPSC carrying α-syn overexpression due to a genomic triplication of the SNCA locus and human embryonic stem cells (hESC)-overexpressing α-syn derived neurons exhibit a decrease in calcium-induced PTP threshold opening, which can lead to increased vulnerability and cell death (Flierl et al., 2014; Ludtmann et al., 2018) (Figure 2).

As seen previously stated, ROS generated by α-syn affects multiple mitochondrial elements; in this case, ROS can trigger apoptosis due to oxidizing and stimulating of PTP elements. Additionally, α-syn can lower the threshold of activation of PTP, which increases these phenomena. This pathological mechanism may be one of the pathways through which α-syn can lead to mesencephalic dopaminergic neurons to death.

For all the above, mitochondria have been postulated as the central organelle affected in synucleinopathies, although, as will be discussed in the upcoming sections, other organelles could also play a fundamental role in the pathological development.

## Nuclear Localization of Alpha-Synuclein Affects Transcriptional Regulation

The name *syn-nuclein* indicates synaptic and nuclear distribution (Maroteaux et al., 1988). Although the synaptic function of α-syn is widely studied, particularly the mechanisms involved in vesicular transport (Hasegawa et al., 2017), its function within the nucleus is poorly understood. Interestingly, α-syn appears to modulate the physical properties of DNA. Using WT α-syn on nanofluids with DNA, Jiang et al. (2018) found that its binding was, to a large extent, driven by electrostatic interactions and gradually stiffened as α-syn was binding to naked DNA, resulting in gradual increase in DNA length.

One of the key aspects of the interaction between α-syn and the nucleus is its importation from the cytoplasm. A recent report has described the critical role of the nuclear pore complex for the mobilization of α-syn through the nuclear membrane. By tagging both α-syn and karyopherin alpha 6 (a nuclear adaptor protein) in SH-SY5Y cells and mouse fetal primary cortical neurons, and evaluating their physical interaction using FRET, it was found that α-syn interacts with karyopherin alpha 6 after α-syn has been sumoylated, a mandatory process for its transport to the nucleus (Ryu et al., 2019).

Furthermore, various transcripts of α-syn with different deletions were expressed on PC-12 cells and followed through its mobilization from the cytoplasm to the nucleus. With this model, Ma et al. (2014) found that the C-terminus of α-syn (residues of amino acids 103–140) appeared to play an important role in its nuclear accumulation (Ma et al., 2014). Apparently, the protein forms a specific spatial structure based on C-terminus interactions with the central part, necessary to join together; this may allow for the nuclear accumulation of α-syn. Moreover, the nuclear accumulation of α-syn is mediated by importin alpha, but the results obtained suggest that an unknown protein mediates the direct interaction between α-syn and importin (Ma et al., 2014). No further information regarding the missing protein has been reported yet.

The interactions that α-syn can have in the nucleus are not restricted to DNA. It has been found that α-syn fibrillation is accelerated in the presence of histone H1 and core histones. In addition, Paraquat-induced injury in mice increases α-syn concentration in the nuclei of the midbrain (Goers et al., 2003). Indeed, the signal of histone H1 colocalized with α-syn and also with H3 and NeuN (neuronal nuclei marker). Therefore, it was suggested that the colocalization observed might be an indication of a complex formation between α-syn and histone H1 and/or H3 in the nucleus (Goers et al., 2003).

Histone condensation is largely responsible for the formation of heterochromatin and euchromatin, the first being transcriptionally repressed and the latter being transcriptionally active. This transcription apparatus is partially dictated by biochemical changes such as acetylation, methylation, or phosphorylation on DNA itself or on histone residues. When the charge of the histone tails is changed by these modifications, they can either lose or gain affinity for DNA and for other histones, allowing or restricting the entrance of transcription factors and other transcription-associated proteins. The proteins responsible for “writing” these marks are, among others, DNA methyltransferases, histone acetyltransferase, and histone methyltransferases, and its corresponding “erasers” are histone deacetylases (HDACs). An extensive review on epigenetic markers can be found in Huang et al. (2014).

Alpha-synuclein interacts with epigenetic “writers.” It has been observed that in transgenic flies expressing hWT α-syn ubiquitously under control of the daG32-GAL4 promoter, an increase in histone-H3 lysine-9 (H3K9) methylation was present (Sugeno et al., 2016). Furthermore, by overexpressing hWT α-syn in rat B103 neuronal cells, Desplats et al. (2011) found that α-syn can retain DNA methyltransferase 1 in the cytoplasm of neuronal cells compromising DNA methylation (Figure 3). Indeed, lower levels of methylation were found in intron 1 of the *SNCA* gene (Desplats et al., 2011). Moreover, in dopaminergic-like cells differentiated from SH-SY5Y cells with inducible hWT α-syn expression, higher levels of H3K9 mono- and dimethylation were observed (Figure 3), probably affecting mRNA expression of the neural cell adhesion molecule L1CAM and the synaptosomal-associated protein SNAP25, as well as H3K9me2 at the SNAP25 promoter. This might act within a feedback network to tone the release of synaptic vesicles, and this mechanism could derail and contribute to synaptic dysfunction occurring in PD (Sugeno et al., 2016).

Alpha-synuclein can also interact with epigenetic “erasers.” Substantial evidence has shown that α-syn restricts and maintains HDACs on the cytoplasm, inhibiting its normal function. Indeed, human mutant p.A30P and p.A53T α-syn in SH-SY5Y cells bind to histones, which in turn lowers histone acetylation, a key mark for transcriptional activation (Figure 3). Consequently, inhibiting HDACs protects against α-syn neurotoxicity, as probed in transgenic flies and in SH-SY5Y cells (Kontopoulos et al., 2006).

Alpha-synuclein is heavily involved with histone deacetylase 4 (HDAC4), another important epigenetic eraser that is highly expressed in neurons and forms part of the HDACs class IIa that can shuttle between the cytoplasm and nucleus (Takahashi-Fujigasaki and Fujigasaki, 2006). Indeed, by exposing p.A53T-mutant α-syn mice or PC-12 cells to a sub-toxic concentration of MPTP (1-methyl-4-phenyl-1,2,3,6-tetrahydropyridine), HDAC4 nuclear accumulation was induced. Furthermore, HDAC4 localized in the nucleus and mediated cell death in p.A53T cells by repressing the transcriptional activity of CREB (cAMP response element binding protein) and myocyte enhancer factor 2A (MEF2A) (Wu et al., 2017).

Finally, on H4 cells, it has been reported that the pS129-α-syn form has an affinity for the nucleus, down-regulating important cell-cycle genes like *CCNB1* and *E2F8* (Pinho et al., 2019), indicating a plausible effect on progression between cell-cycle phases. In addition, Schaser et al. (2019) showed that pS129-α-syn is rapidly recruited to laser-induced DNA damage sites in the nucleus of *in vivo* mouse brain cells, as well as in a mouse primary cortical neuron system, having a plausible role on double-strand break repair (Schaser et al., 2019). This pathological form has also been observed within the nucleus of neurons in brain areas such as the basolateral amygdala, cortex, and hippocampus from aged p.A30P α-syn mice with impaired cognitive behavioral phenotypes. In contrast, these changes were not found in healthy young p.A30P α-syn mice (Schell et al., 2009). Thus, phosphorylation of α-syn might also be linked to an aging process.

In summary, although α-syn physiological role within the nucleus has not yet been completely enlightened, the study of its interactions with nuclear regulatory elements has re-emerged in light of the current advances concerning epigenetic mechanisms, particularly the pathological repercussions involving PD. Indeed, α-syn appears to affect transcriptional regulation by physically interacting with both epigenetic writers and erasers. This can result in its own transcriptional up-regulation, thus promoting accumulation and, in consequence, α-syn aggregation within the cell. This in turn affects the normal function of other organelles compromising cell viability. Therefore, searching for mechanisms by which α-syn transcription can be down-regulated to avoid its aggregation may prevent or diminish neuronal death.

## Alpha-Synuclein and Organelles Involved in Vesicular Trafficking

Another mechanism involved in the synucleinopathies includes vesicular trafficking, a critical system for cellular architecture and communication between organelles within the cell (Vassilieva and Nusrat, 2008; Brady et al., 2012). It is important to highlight that the main organelles that participate in this process are the ER, the GA, and the endolysosomal system.

### Alpha-Synuclein Can Induce Stress in the Endoplasmic Reticulum

The main function of the ER is to synthesize both lipids and proteins, as well as protein folding and post-translational modification of proteins for protein maturation. Furthermore, the ER works as an intracellular Ca^2+^ reservoir that maintains an oxidative environment and the adequate micromolar concentration of free Ca^2+^ (0.05–0.1 μM) (Calì et al., 2011; Chen et al., 2013; Kramer, 2016). The ER has also been implicated in sensing the quantity of misfolded or unfolded proteins when there is an insufficiency in the protein-folding capacity of the ER. Hence, in an increase in misfolded proteins, the ER activates the system of unfolded protein response (UPR) and regulates the correct folding or the degradation of proteins (Walter and Ron, 2011).

In experiments with yeasts, the UPR system is activated in basal conditions but, under the overexpression of α-syn, leads to the overactivation of the UPR inducing ER stress, whereas, in SH-SY5Y cells, the pS129-α-syn causes cell death through the overactivation of the UPR system (Cooper et al., 2006; Sugeno et al., 2008) (Figure 3). In hiPSC-derived neurons, there are no impairments shown in the ER. In contrast, when α-syn is overexpressed due to a *SNCA* genomic triplication, ER stress has been observed. This promotes the activation of the UPR through the induction of the IRE1α/XBP1 (inositol-requiring transmembrane kinase/endoribonuclease 1α/X-box binding protein 1) pathway, which is a signaling cascade involved in survival signaling under the UPR condition (Table 1) (Heman-Ackah et al., 2017). This system is initiated by IRE1α activation, which cleaves XBP1 mRNA leading to its transcriptional function. XBP1 then binds to unfolded protein response element (UPRE) and up-regulates genes encoding ER chaperones to enhance the folding efficiency and the degradation mechanism (Yoshida et al., 2001; Takayanagi et al., 2013; Jiang et al., 2016; Jiao et al., 2017). The process mentioned before is important due to the accumulation of poly-ubiquitinated proteins like α-syn and other cytoskeleton proteins in histopathological brain studies from PD patients, where UPR was found to be active in response to ER stress (Conn et al., 2004; Hoozemans et al., 2007; Colla, 2019). This indicates that this function could be induced to prevent the cytotoxicity associated with ER stress and unfolded proteins (Hoozemans et al., 2007).

In recent studies, it has been demonstrated in dopaminergic (CATH.a) and PC-12 cells, the expression of ER stress markers like GRP78 (78-kDa glucose-regulated protein, chaperone), IRE1α, and phosphorylated eukaryotic initiation factor 2α (peIF2α) is present in basal conditions. However, the overexpression α-syn mutant variants (p.A53T or p.A30P) in cells leads to an increase of these ER stress markers, resulting in apoptosis caused by the loss of the calcium homeostasis, through the action of stress-induced proteins like Herp (homocysteine-induced ER protein) (Smith et al., 2005; Belal et al., 2012; Yoon et al., 2018). Likewise, the aggregates of α-syn, but not the monomers, can bind and activate SERCA, decreasing the cytosolic Ca^2+^ concentration that leads to cellular apoptosis in *C. elegans* and SH-SY5Y cells (Betzer et al., 2018) (Figure 3). On the other hand, the expression of α-syn mutants (p.A53T or p.A30P) promotes its oligomerization in transgenic mice. These oligomeric forms are involved in the pathogenesis of PD, and they could be accumulated within the ER by chronic stress in this organelle (Colla et al., 2012).

Mitochondria-associated ER membranes (MAM) are interconnected by a section from the mitochondrial surface that is proximal to the ER membrane (Hayashi et al., 2009). MAM can regulate cellular processes that directly communicate mitochondria and the ER like calcium homeostasis, lipid metabolism, ATP production, transport and biogenesis of mitochondria, ER stress, response to unfolded proteins, and autophagy (Paillusson et al., 2017). A recent study in BE(2)-M17 neuroblastoma cells showed that mutations in p.A53T or p.A30P can lead to α-syn re-location from the cytoplasm to the vicinity of MAM (Guardia-Laguarta et al., 2014), mainly altering calcium homeostasis that results in neuronal cytotoxicity (Calì et al., 2011). Moreover, Grassi et al. (2018) found that pS129-α-syn colocalized with Tom20 (a marker of mitochondria) and GRP78, which means that it was localized in the vicinity of MAM. According to these authors, it could prevent the fusion of the mitochondria, as well as alter functions from the ER.

*ELO1*, *ELO2*, and *ELO3* are genes that code to three lipid elongases that are involved in fatty acid elongation within the ER (Jakobsson et al., 2006). In *Saccharomyces cerevisiae*, these genes promote a regular growth, while null mutations in these genes show normal growth. Additionally, the null expression of *ELO1*, *ELO2*, and *ELO3* concomitant to the expression of hWT α-syn, p.A53T, or p.E46K through plasmids in this model showed a defect in cellular growth, as well as aberrant vesicular traffic from the ER to the GA, and ROS accumulation in the cytosol (Lee et al., 2011), thus indicating that the inhibition in the synthesis of very long fatty acids promotes the toxicity of α-syn (Lee et al., 2011).

Recently, Paiva et al. (2018) found that mice with normal expression of α-syn did not present changes in transcriptional regulation. Nevertheless, transgenic mice with expression of hWT α-syn and the p.A30P mutation showed 18 and 2165 genes deregulated, respectively, compared to control mice that just expressed mouse α-syn. In the context of the ER, *COL4A2* is an important gene that has been found up-regulated. This gene has also been related to increased levels of ER stress when it has an abnormal expression. In the same study, Lund human mesencephalic (LUHMES) cells that express hWT α-syn did not present significant *COL4A2* expression. However, LUHMES cells with p.A30P mutation showed ER stress, reflected in an increase in levels of *COL4A2* and *CALNEXIN* (Paiva et al., 2018).

Alpha-synuclein is also implicated in ER-GA vesicular trafficking, regulated by the small guanosine triphosphatases (GTPase) called Rabs. Rabs are hydrolase enzymes that produce guanosine diphosphate (GDP) by the hydrolysis of guanosine triphosphate (GTP), being an important molecular switch in vesicular traffic (Allan et al., 2000). In hiPSC-derived midbrain dopaminergic neurons, Rab1A is localized in the ER-GA proximity near the perinuclear position, but when hWT α-syn is overexpressed using a lentivirus, Rab1A has a diffuse localization with an ER-GA fragmentation and disruption of the ER-GA traffic. However, the mechanism by which this occurs has not been studied (Mazzulli et al., 2016).

At the same time, retention in ER sorting receptor 1 (RER1) is a protein involved in the retention of proteins in the ER and retrieval of ER membrane proteins from the GA (Füllekrug et al., 1997), also mediating ER-Golgi trafficking (Sato et al., 1997). Recently, Park et al. (2017) found that α-syn interacts with RER1 and showed that overexpression of RER1 can reduce α-syn levels of HEK293 in H4 cells. Park et al. (2017) also found that α-syn is ubiquitinated by NEDD4, an E3 ligase, which interacts with RER1 to induce α-syn degradation by the proteasome system (Chung et al., 2013). Furthermore, in histopathological brain studies from DLB, RER1 has been located in LB-like round structures, presenting colocalization with pS129-α-syn, unlike control brains (Park et al., 2017); indicating the relevance of RER1 in regulating α-syn activity during vesicular trafficking.

For all the above, it is clear that α-syn overexpression, its mutants or pathological forms, can induce stress in the ER conducting at its malfunctioning. Furthermore, growing evidence indicates that α-syn interacts with Rab1 and RER1. Moreover, RER1 appears to regulate α-syn levels. Thus, by elucidating the role of RER1 with α-syn and how to regulate its levels, α-syn cellular concentrations could also be regulated.

### Alpha-Synuclein Induces Golgi Apparatus Malfunction

The GA is an organelle formed by a membranous cisterna, in which the proteins emerging from the ER are fully processed and sorted to different cellular destinations. The series of cisternae is divided into *cis*, medial, and *trans* Golgi compartments, assembling the *cis* Golgi network (CGN) and the *trans* Golgi network (TGN). In the CGN, proteins undergo modifications, whereas in the TGN, the modified proteins are packed and sorted for delivery to the lysosomes, plasma membrane, or secretory vesicles (Malhotra and Mayor, 2006; De Matteis and Luini, 2008; Huang and Wang, 2017).

The specific physiological functions of α-syn in GA are still unknown. However, the GA has been shown to have impaired ionic transport and membrane traffic (Fan et al., 2008) as well as deficits of axonal transport associated to GA fragmentation (Fujita et al., 2006; Fan et al., 2008).

Among the observed alterations of the GA on synucleinopathies is the calcium-transporting ATPase 1 (PMR1) pump, which regulates intracellular levels of Ca^2+^ and Mn^+2^ ions in the GA (Dürr et al., 1998). In neurotoxic conditions such as α-syn accumulation, a decrease in cell death has been observed due to the prevention of the intracellular Ca^2+^ overload, a mechanism performed by the PMR1 pump in *C. elegans* (Kourtis et al., 2012; Nikoletopoulou and Tavernarakis, 2017). Nonetheless, in models of PD (yeast, flies, and nematodes), PMR1 has been linked to α-syn toxicity by increasing Ca^2+^ levels and the depletion of PMR1 decreases cell death (Büttner et al., 2012). Thus, PMR1 could have significant relevance in the development of the α-synucleinopathy as a therapeutic target, although more research on mammal models for PMR1 is required in order to develop any type of treatment.

Regarding the role of α-syn in GA fragmentation, Liu et al. (2018) found GA with an apparent breakdown and diffusely distributed in primary rat astrocytes that had an overexpression of WT α-syn or p.A30P and p.A53T mutants. Furthermore, in the study described in the ER section, Paiva et al. (2018) found that control LUHMES cells (constitutive expression of α-syn) had a normal morphology of GA in contrast to cells with exogenous expression of hWT α-syn and the p.A30P mutation that showed GA fragmentation. However, the percentage with diffuse morphology was higher in cells with the p.A30P mutation compared to hWT α-syn. Additionally, in a study in COS-7 cells that express α-syn using a recombinant adeno-associated virus vector (*SNCA* AAV vector), GA morphology was normal in cells with diffuse staining of α-syn, but when the cells had prefibrillar α-syn, fragmentation and dispersion of GA were shown. Indeed, in cells with fibrillar inclusions, there was no apparent association to GA fragmentation, which suggests an early event before fibrillar aggregation of α-syn in COS-7 cells (Gosavi et al., 2002).

A hypothesis for GA fragmentation is the impairment in microtubules that had been related to the maintenance of the structure of this organelle. In this sense, it has been found that in SH-SY5Y cells, the normal juxtanuclear structure of GA was altered in a similar pattern in both cells exposed to drug-induced depolymerization of microtubules and in cells with α-syn overexpression. Additionally, α-syn overexpression affected the transport mechanism dependent on microtubules (Lee et al., 2006).

Furthermore, other studies on GA fragmentation have found some alterations and possible interactions of α-syn. Gosavi et al. (2002) found that in contrast to COS-7 cells with constitutive expression of α-syn, and in conditions of increased levels of monomeric α-syn, the COS-7 cells with prefibrillar α-syn had lower levels of dopamine transporter on the cell surface, suggesting an impairment in protein trafficking and maturation in the medial Golgi (Gosavi et al., 2002) (Figure 3).

Another compromised mechanism is autophagy. Indeed, it has been found that, in human neuroblastoma SKNSH cells, the overexpression of α-syn inhibits RAb1A, which promotes mislocalization of the autophagy protein Atg9 in the TGN (an important feature for the autophagosome formation) (Winslow et al., 2010) (Figure 3). Furthermore, astrocytes derived from hESC exposed to oligomers from recombinant hWT α-syn showed accumulation of this form in the TGN. Interestingly, no GA fragmentation was observed (Rostami et al., 2017). In addition, due to the fact that most of the autophagosome membranes come from ER and GA, Rostami et al. (2017) studied various steps of autophagy pathway, like formation of conjugated LC3B, LC3BII/I ratio, and expression of p62. They observed that α-syn oligomers disrupted autophagosome/lysosome function, suggesting that the accumulation of α-syn aggregates in the TGN could impair the autophagosomal and the mitophagy pathways.

Alpha-synuclein has also been related to function as an adaptor protein by linking to proteins involved in vesicular transport (Fan et al., 2008). In fact, in a toxicity study in yeast through overexpression of α-syn, it was reported that mutations in genes involved in vesicular traffic and in lipid metabolism increased lethality of α-syn overexpression, which led to suggest that these genes are the primary pathway that regulates toxicity of α-syn (Willingham et al., 2003). Another evidence of the relation between α-syn and vesicular traffic was presented by Soper et al. (2008) using a model of *S. cerevisiae* with inducible expression of hWT α-syn or the p.A53T mutant. They observed accumulation of secretory and transport vesicles from ER to GA, and α-syn appeared to colocalize around the vesicles.

In addition, a study of overexpression of hWT α-syn and the p.A53T mutant in yeast found an interruption in vesicular trafficking from the ER to the GA, although p.A53T induced faster blockage of vesicular trafficking than hWT α-syn (Cooper et al., 2006) (Figure 3). Interestingly, the time of the impairment in vesicular transport corresponded to the time of impairment of growth. Furthermore, the same study found that overexpression in genes promoting transport from the ER to the GA like Rab protein Ypt1p, the SNARE [soluble NSF (N-ethylmaleimide-sensitive factor) attachment protein receptor] protein, Ykt6b, the ubiquitin protease Ubp3p, etc., suppresses α-syn toxicity. Additionally, they found that Ypt1p was frequently localized in α-syn cytoplasmic inclusions. This suggested that the cytotoxic form of α-syn might associate with transport vesicles, as α-syn usually does with synaptic vesicles (Cooper et al., 2006). In accordance with Soper et al. (2011), they found that in yeast with expression of hWT α-syn, α-syn was colocalized with Rabs involved in retrograde endosome-Golgi transport (Ypt6p), intra-Golgi (from *cis* to *trans* Golgi side), and post-Golgi trafficking (Ypt31p, Ypt32p). However, they did not find direct interaction between Rabs and α-syn. However, they showed the ability of α-syn to induce accumulation and mislocalization of some Rab proteins (Soper et al., 2011).

Besides, Gitler et al. (2008) found a normal localization of Rab fusion proteins in WT yeast compared with expression of one copy of α-syn yeast, while yeast with expression of two copies of α-syn had an alteration in the localization of the Rabs Ypt1, Ypt31, Sec4, Ypt6, Vps21, and Ypt52, and was colocalized with α-syn. Also, it was found that α-syn did not alter vesicle buds and their target to cell periphery; however, α-syn avoided the docking and/or the fusion of vesicles. Moreover, due to evidence that Rab1 and Ypt1 rescue dopaminergic neurons of α-syn toxicity in *Drosophila*, *C. elegans*, and primary cultures of rat midbrain neurons (Cooper et al., 2006; Gitler et al., 2008), it has been suggested that trafficking impairment starts at the ER and proceeds to the GA (Gitler et al., 2008).

It has been observed that, for toxic inclusions, formed by α-syn-positive vesicular clusters that colocalize with protein markers of several trafficking routes, a great proportion of this protein is phosphorylated. Interestingly, by blocking α-syn phosphorylation in yeast, there is an augmentation in the toxicity observed that affect cellular trafficking (Sancenon et al., 2012). In addition, Tenreiro et al. (2014) found that the accumulation of hWT α-syn induced an autophagic response. In contrast, the mutant phosphorylated form (pS129-α-syn) showed paired activity in the same pathway, reducing aggregation levels (Tenreiro et al., 2014). Interestingly, they also observed that the expression of two unphosphorylated mutants, S129A-α-syn and S129E-α-syn, promoted the formation of α-syn toxic inclusions and diminished the capacity of the cell to eliminate them (Tenreiro et al., 2014). Therefore, blocking α-syn phosphorylation promotes cytotoxicity in yeasts.

Finally, a study carried out with rat kidney epithelial (NRK) cells with different ranges of expression of hWT α-syn showed that at low expression levels, it was associated with minimal inhibition of transport from the ER to the GA, while high levels of expression increased inhibition proportionally (Thayanidhi et al., 2010). Meanwhile, in cells with expression of the p.A53T α-syn mutant, the inhibition was almost complete in low expression. In addition, in rat dopaminergic neuroendocrine (PC12) cells, the expression of the p.A53T α-syn mutant showed lower transport inhibition (around 30%) compared to NRK cells (around 50%), maybe due to the existence of a protection mechanism or factor against α-syn (Thayanidhi et al., 2010). The same group found that the p.A53T α-syn mutant caused antagonisms of α-syn and SNAREs, which are part of the trafficking machinery from the ER to the GA and could be the cause of delay in transport. Also, α-syn inhibits the pre-Golgi coat protein complex II vesicle docking and fusion *in vitro*. An extensive review about α-syn and membrane trafficking can be found in Hasegawa et al. (2017).

### The Endolysosomal System Is Related to Accumulation of Alpha-Synuclein

The endolysosomal system regulates vesicle traffic and it is involved in proteostasis (Winckler et al., 2018). Several proteins seem to have relevance in α-syn accumulation in the endolysosomal system. One of them is the endosome and lysosome transmembrane protein ATP13A2, which suffers a mutation known as Dup22 (a duplication of 22 pair bases), resulting in a truncated protein presenting 6 of 10 transmembrane domains. With this truncated protein, the aggregation of α-syn and ER stress is promoted in H4 neuroglioma cells, while the depletion of ATP13A2, using a short hairpin RNA, promotes the aggregation of α-syn, leading to cell death by decreased lysosomal activity in primary cortical neurons (Usenovic et al., 2012; Lopes et al., 2016).

Another molecule that may promote α-syn accumulation is vacuolar protein sorting-associated protein 35 (VPS35). VPS35 is a component of the retromer complex involved in the transport of the endosome to the TGN, the membrane protein retrieval, the endosomal recycling (Yun et al., 2017), and a rare form of autosomal-dominant parkinsonism (Vilariño-Güell et al., 2011). It has been shown in mice with a VPS35 deletion that oligomeric and phosphorylated α-syn is increased. Furthermore, it was found that intracellular α-syn is increased in tyrosine hydroxylase (TH, the rate-limiting enzyme of catecholamine biosynthesis) positive neurons of the SN *pars compacta* (Tang et al., 2015). In the same model, the expression of Lamp2a, a receptor of chaperone-mediated autophagy (CMA), decreases, suggesting unpaired α-syn degradation via CMA, as a result of an alteration in lysosomes, due to a deregulation of Lamp2a (Tang et al., 2015). Moreover, in transgenic mice with α-syn overexpression, it was localized in the lysosomal lumen (Mak et al., 2010). In addition, an increase of Lamp2a was observed in nigral dopaminergic neurons also with α-syn overexpression, which indicates that the CMA is induced to clear the excess of α-syn (Berry et al., 2010; Mak et al., 2011).

To help in the TGN tasks, the GGAs proteins (Golgi-localized, gamma adaptin ear-containing, ARF-binding), a group of coat proteins, are recruited from the cytosol onto the TGN, where they have the function of protein transport to the endosome/lysosome system (Ghosh and Kornfeld, 2004). In this sense, when N2A and HEK293 cells overexpress GGAs proteins, they showed an increase in oligomeric α-syn secreted to the medium (Einiem et al., 2017). The role of GGAs in the secretion of oligomeric α-syn makes it interesting for the study of propagation of α-syn, considering the conjecture of PD as a prion disease (see section “Introduction”).

Interestingly, the overexpression of α-syn in COS-7 and SH-SY5Y cells showed that just the oligomers of α-syn and not the fibrillary form can be degraded by the lysosomal system, while the inhibition of the lysosome causes the accumulation of the aggregates of α-syn (Lee et al., 2004). Recently, in the experiment of Mazzulli et al. (2016), the hiPSC-derived midbrain dopaminergic neurons showed a basal proteolysis rate using leupeptin, which is a protease inhibitor of enzymes in lysosomes. Nonetheless, the overexpression of hWT α-syn by a lentivirus produced a lysosomal dysfunction, which declined the proteolysis rate showing increased lysosomal mass and a reduction of the hydrolase activity. Lysosomal enzymes such as cathepsin B and hexosaminidase showed a significant reduction of their activity; this was also observed in non-lysosomal enzymes like β-galactosidase in the GA when hWT α-syn was overexpressed. The reduction of this activity was due to defects in vesicular traffic. The results from the experiments described were confirmed by measuring the hydrolase mature:immature rate of hexosamine A and B. Both proteins acted in the lysosome and the result revealed enzyme accumulation in ER-GA vesicles, implying that the accumulation of α-syn was altering the lysosomal degradation by interrupting vesicular traffic between the ER and the GA (Mazzulli et al., 2016).

In summary, dysfunction in vesicular traffic and impairments in membranous organelles, such as the synthesis of proteins and lipids in the ER, post-translational modifications, and sorting and delivery of vesicles by the GA and the endolysosomal system, can be a result of the accumulation of α-syn. Concurrently, the accumulation of α-syn leads to cellular impairment and damage of the homeostatic balance. Therefore, it is not clear if α-syn accumulation is the first step of cytotoxicity or if it is a consequence of impairments in these organelles.

## Concluding Remarks

The last two decades have been critical in gaining knowledge about α-syn function, in part due to the development of *in vitro* and *in vivo* models that, despite not replicating PD entirely, resemble pathological processes in humans (e.g., transgenic mouse models, human stem cell modeling), especially in the context of PD and other synucleinopathies. α-syn has a fundamental role, given its multiple interactions in several subcellular compartments and through the multiple conformations (monomer, oligomer, and fibril conformation), as well as its several post-translational modifications, which generate a spectrum of adverse effects for the cell that can exacerbate and finally culminate in cell death. At the same time, some physiological roles of α-syn have been also enlightened, especially in bioenergetic control, as well as vesicle transport from the GA to the ER, which outlines the importance of this protein for its cellular function. Despite these advances, the role of α-syn in early events of the disease process remains to be determined, including developmental and differentiation events of dopaminergic neurons. This will uncover early biomarkers and even advance therapeutic strategies to modify the natural history of the disease.

## Author Contributions

LB-C and RA-S: analysis of mitochondrial-alpha synuclein interactions and overview of prionic diseases. RR-A and IM-M: implications of synuclein with nucleus and DNA. MR-H: description of the interactions of alpha-synuclein with soluble proteins and endolysosomal system. AB-O: review of alpha synuclein and organelles involved in vesicular trafficking. BS: analysis, writing modifications, and feedback through all the text. MG-C: supervision of research program, writing, analysis and interpretation throughout all the text, and editing of the manuscript.

## Conflict of Interest

The authors declare that the research was conducted in the absence of any commercial or financial relationships that could be construed as a potential conflict of interest.

## Abbreviations

α-syn: Alpha-synuclein
CMA: chaperone-mediated autophagy
COXII: cytochrome C oxidase subunit 2
DLB: dementia with Lewy bodies
ER: endoplasmic reticulum
ETC: electron transport chain
FRET: fluorescence resonance energy transfer
GA: Golgi apparatus
GGAs: proteins, Golgi-localized gamma adaptin ear-containing ARF-binding
H3K9: histone-H3 lysine-9
HDACs: histone deacetylases
Herp: homocysteine-induced ER protein
hESC: human embryonic stem cells
hiPSC: human-induced pluripotent stem cells
hWT: human wild-type
IMM: inner mitochondrial membrane
IRE1 α /XBP1: inositol-requiring transmembrane kinase/endoribonuclease 1 α /X-box binding protein 1
LB: Lewy bodies
LUHMES: Lund human mesencephalic
MEF2A: myocyte enhancer factor 2A
MSA: multiple system atrophy
mtDNA: mitochondrial DNA
MTS: mitochondrial targeting signal
NAC: non-amyloid component
NDGA: nordihydroguaiaretic acid
OMM: outer mitochondrial membrane
OXPHOS: oxidative phosphorylation
PD: Parkinson’s disease
peIF2 α: phosphorylated eukaryotic initiation factor 2 α
PLA: proximity ligation assay
PMR1: calcium-transporting ATPase 1
pS129- α-syn: alpha-synuclein phosphorylated at serine 129
PTP: permeability transition pore
pY125- α-syn: alpha-synuclein phosphorylated at tyrosine 125
ROS: reactive oxidative species
SERCA: sarco/endoplasmic reticulum Ca^2+^-ATPase
SN: substantia nigra
TGN: *trans* Golgi network
TH: tyrosine hydroxylase
TOM complex: translocase of the outer membrane
UPR: unfolded protein response
UPRE: unfolded protein response element
VDAC: voltage-dependent anion channel
VPS35: vacuolar protein sorting-associated protein 35.
